# Total Intravenous Anesthesia Compared to Inhalational Anesthesia in Patients Undergoing Arthroscopic Rotator Cuff Repair

**DOI:** 10.7759/cureus.50775

**Published:** 2023-12-19

**Authors:** Christopher Rennie, Katerina N Futch, Jane C Brennan, Benjamin M Petre, Sohail Zaidi, Justin J Turcotte, Andrea H Johnson, Daniel E Redziniak

**Affiliations:** 1 Dr. Kiran C. Patel College of Osteopathic Medicine, Nova Southeastern University, Tampa, USA; 2 Orthopedic Research, Anne Arundel Medical Center, Annapolis, USA; 3 Orthopedic Surgery, Anne Arundel Medical Center, Annapolis, USA; 4 Anesthesiology, Anne Arundel Medical Center, Annapolis, USA; 5 Orthopedics, Anne Arundel Medical Center, Annapolis, USA

**Keywords:** postoperative outcomes, narcotic use, postoperative pain, inhalational anesthesia, total intravenous anesthesia (tiva), arthroscopic rotator cuff repair

## Abstract

Background

Inhalation anesthesia (IA) and total intravenous anesthesia (TIVA) are common general anesthesia techniques. During rotator cuff repair (RCR), an interscalene block is beneficial for intraoperative and early postoperative pain control. This study aimed to evaluate postoperative outcomes and opioid usage in patients undergoing arthroscopic RCR with an interscalene block and either IA or TIVA.

Methodology

A retrospective observational study was performed of 478 patients undergoing RCR at a single institution. Demographics, surgical details, intra and postoperative medications, and 90-day outcomes were collected. Univariate and multivariate analyses were performed to evaluate differences between groups.

Results

In total, 309 (64.6%) patients received IA and 169 (35.3%) received TIVA. Patients receiving IA were more likely to have comorbidities, such as diabetes (p = 0.002), sleep apnea (p = 0.006), gastroesophageal reflux disease (p < 0.001), and hypertension (p < 0.001). After adjusting for differences between groups in the multivariate analysis, patients who received TIVA had significantly shorter surgical time (β = -14.85, p < 0.001) and perioperative time (β = -21.01, p < 0.001) and significantly lower first post-anesthesia care unit Pasero opioid-induced sedation scores (β = -0.022, p = 0.040). Patients who received TIVA were less likely to receive intraoperative narcotics (odds ratio = 0.38; p = 0.031).

Conclusions

TIVA appears to be a safe and effective anesthetic for patients undergoing arthroscopic RCR. TIVA is a potentially beneficial alternative to IA for this patient population.

## Introduction

Rotator cuff injuries are a common orthopedic condition, affecting over 25% of all people over the age of 60 and nearly 50% of those over the age of 70 [1,2]. Rotator cuff repairs (RCRs) are one of the most commonly performed orthopedic procedures, with over 250,000 performed each year in the United States alone [1]. RCR is a safe and effective surgical procedure that benefits individual patients and the wider society [3,4]. However, RCR is often associated with significant postoperative pain and opioid use; the choice of anesthetic technique used can influence patient outcomes and is an important consideration [5,6]. General anesthesia with an endotracheal tube is typically the standard technique used in RCR due to several factors, including patient positioning and facilitating hypotensive anesthesia to minimize intraoperative blood loss [6]. In patients undergoing RCR, the use of peripheral nerve blocks is proven to be a beneficial adjunct to general anesthesia (GA) for both intraoperative and early postoperative pain control; interscalene blocks are one of the most effective peripheral nerve blocks in RCR [6-8].

The most commonly used forms of GA in these procedures are inhalational anesthesia (IA) and total intravenous anesthesia (TIVA) [9]. Historically, IA has been used as the standard form of GA as it acts on GABA receptors to induce central nervous system (CNS) depression, although it may also induce myocardial and respiratory depression [9,10]. The use of TIVA is becoming more common as a method of GA as it is known for its rapid anesthetic effects and comparatively quicker recovery period; TIVA has the potential for decreased opioid use, decreased nausea, and increased patient satisfaction [11,12]. The most commonly used TIVA agents include propofol, ketamine, and etomidate; these agents are administered intravenously through their effect on various GABA and adrenergic receptors to quickly induce anesthesia [9,13]. Within the fields of anesthesiology and orthopedic surgery, there is limited data regarding the potential differences in clinical outcomes for patients undergoing RCR based on the form of GA used [14,15]. The primary objective of this study was to compare intra and early postoperative opioid use in patients undergoing RCR via IA or TIVA. Secondary objectives included comparing concomitant intra and postoperative medications used, short-term postoperative outcomes, and 90-day emergency department (ED) visits and admissions.

## Materials and methods

Study population and setting

This study was deemed exempt by the institutional review board. A retrospective observational study of patients undergoing primary arthroscopic RCR from January 2019 through February 2022 was conducted. Procedures were performed by one of nine board-certified orthopedic surgeons in a single hospital-based outpatient surgery center. Patients were excluded from the study if they underwent open RCR, revision RCR, or if they did not receive a preoperative interscalene peripheral nerve block.

Perioperative protocol

All surgeries were performed on an outpatient basis under either inhaled or total intravenous GA. All patients received an interscalene peripheral nerve block performed preoperatively. The peripheral nerve blocks were performed with consistent technique among anesthesiologists with 20 mL of ropivacaine 0.5%. The IA protocol consisted of preoperative sedation using a combination of midazolam, fentanyl, and/or propofol, followed by induction and airway management via laryngeal mask airway (LMA) or endotracheal tube. Anesthesia was maintained using inhalational anesthetics, most commonly sevoflurane. The TIVA protocol consisted of preoperative sedation using a combination of midazolam, fentanyl, and/or propofol, followed by induction and maintenance of GA through a continuous intravenous infusion of propofol and oxygen delivered typically by nasal cannula with the occasional use of LMA as appropriate. Additional medications for pain and nausea were used at the discretion of the anesthesiologist. All medication dosages were determined by the anesthesiologist as appropriate for the individual patient. All surgical procedures were performed in the beach chair position.

The Pasero opioid-induced sedation scale (POSS) is a scoring system commonly used in post-anesthesia care units (PACUs) to evaluate sedation. Patients are scored from 1 to 4, with 1 being the most alert and 4 being somnolent and unresponsive; 1 and 2 are considered acceptable scores, and 3 and 4 require intervention [16].

Data collection and analysis

Patient demographics, comorbidities, intraoperative and postoperative medications, and surgical and PACU details were manually recorded from the electronic medical record. Ninety-day hospital returns, including ED visits and hospital admissions, were obtained using the Chesapeake Regional Information System for Our Patients (CRISP) and accounted for visits to both our institution and outside regional institutions. The primary endpoints were the need for narcotics and the total milligram morphine equivalent (MME) required intraoperatively and in the PACU. Secondary endpoints included differences in nausea, pain, sedation scores, operational throughput measures, and complications resulting in 90-day hospital returns. Univariate statistics including two-sided independent and paired-sample t-tests and chi-square tests were used to evaluate differences in demographics, comorbidities, intraoperative and postoperative medication usage, short-term postoperative outcomes, and hospital returns. Multivariate analysis was performed to evaluate the effect of TIVA on outcomes that demonstrated significant differences at α ≤ 0.10 on univariate analysis after controlling for demographic and comorbidities that varied between groups in the univariate analyses. These included first PACU POSS, intraoperative MME, intraoperative narcotics, minutes in OR, perioperative minutes, postoperative MME, and postoperative narcotics. All statistical analyses were performed using R Studio (version 4.2.2, 2009-2023 RStudio, PBC). Statistical significance was assessed at p-values <0.05.

## Results

Of the 487 patients included in this study, 309 (64.64%) received IA, and 169 (35.36%) received TIVA during their RCR. There were no significant differences in age, sex, race, body mass index (BMI), or American Society of Anesthesiologists (ASA) scores between groups. Patients who received TIVA had lower rates of diabetes (1.2% vs. 7.4%, p = 0.002), sleep apnea (1.2% vs. 7.4%, p = 0.006), gastroesophageal reflux disease (GERD) (3.0% vs. 14.9%, p < 0.001) and hypertension (1.2% vs. 23.0%, p < 0.001) than those who received IA (Table 1).

**Table 1:** Patient demographics and comorbidities. P-values <0.05 are in bold. Data are expressed as mean ± SD or n (%). IA: inhalational anesthesia; TIVA: total intravenous anesthesia; ASA: American Society of Anesthesiologists; COPD: chronic obstructive pulmonary disease; CHF: congestive heart failure; CAD: coronary artery disease; ESRD: end-stage renal disease; CKD: chronic kidney disease; GERD: gastroesophageal reflux disease

Patient demographics and comorbidities	IA (n = 309)	TIVA (n = 169)	P-value
Age (years)	57.82 ± 9.69	59.40 ± 9.33	0.079
Black race	47 (15.2)	19 (11.2)	0.288
Sex			0.562
Female	120 (38.8)	71 (42.0)	
Male	189 (61.2)	98 (58.0)	
Body mass index (kg/m^2^)	30.54 ± 5.74	30.48 ± 5.91	0.913
ASA score 3+	81 (26.2)	38 (22.5)	0.429
Diabetes	23 (7.4)	2 (1.2)	0.002
Sleep apnea	23 (7.4)	2 (1.2)	0.006
COPD	4 (1.3)	1 (0.6)	0.801
Liver disease	3 (1.0)	0 (0)	0.497
Asthma	13 (4.2)	1 (0.6)	0.050
Atrial fibrillation	7 (2.3)	1 (0.6)	0.499
CHF	5 (1.6)	1 (0.6)	0.593
CAD	11 (3.6)	2 (1.2)	0.218
ESRD or CKD	2 (0.6)	0 (0)	0.759
GERD	46 (14.9)	5 (3.0)	<0.001
Hypertension	71 (23.0)	2 (1.2)	<0.001

Patients who received TIVA were less likely to receive dexamethasone intraoperatively (71.6% vs. 89.6%, p < 0.001), had a shorter surgical time (110.98 vs. 94.04 minutes, p < 0.001), shorter perioperative time (defined as combined OR and PACU minutes; 157.05 vs. 177.78 minutes, p < 0.001), and had a lower first PACU POSS scores (2.04 vs. 1.72, p < 0.001) than those receiving IA (Table 2).

**Table 2:** Intraoperative and postoperative measures. P-values <0.05 are in bold. Data are expressed as mean ± SD or n (%) IA: inhalational anesthesia; TIVA: total intravenous anesthesia; MME: morphine milligram equivalents; OR: operating room; POSS: Pasero opioid-induced sedation scale; SpO_2_: percentage oxygen saturation; ED: emergency department

	IA (n = 309)	TIVA (n = 169)	P-value
Intraoperative dexamethasone	277 (89.6)	121 (71.6)	<0.001
Intraoperative ketorolac	143 (46.3)	67 (39.6)	0.193
Intraoperative total MME	10.75 ± 4.09	10.83 ± 4.37	0.853
Intraoperative narcotics	295 (95.5)	154 (91.1)	0.089
Postoperative total MME	9.80 ± 4.21	7.92 ± 3.34	0.052
Postoperative narcotics	49 (15.9)	21 (12.4)	0.379
Minutes in OR	110.98 ± 28.03	94.04 ± 22.57	<0.001
Minutes in recovery	66.80 ± 32.99	63.01 ± 25.88	0.167
Perioperative minutes	177.78 ±44.25	157.05 ± 34.93	<0.001
First PACU nausea	20 (6.5)	6 (3.6)	0.529
First PACU pain	0.46 ± 1.58	0.57 ±1.78	0.507
First PACU POSS	2.04 ±1.04	1.72 ± 0.89	<0.001
First PACU SpO_2_	96.48 ± 2.69	96.57 ± 2.07	0.673
Last PACU nausea	14 (4.5)	4 (2.4)	0.586
Last PACU pain	0.69 ±1.61	0.57 ± 1.52	0.429
Last PACU POSS	1.09 ± 0.35	1.03 ± 0.26	0.064
Last PACU SpO_2_	96.78 ± 2.19	96.67 ±1.85	0.545
90-day ED return	14 (4.5)	12 (7.1)	0.335
90-Day Admission	4 (1.3)	4 (2.4)	0.614

After controlling for age, sleep apnea, diabetes, asthma, GERD, and hypertension, patients who received TIVA had significantly shorter surgical times (β = -14.85, p < 0.001), perioperative times (β = -21.01, p < 0.001), and significantly lower first PACU POSS scores (β = -0.022, p = 0.040). Additionally, those who received TIVA were 2.6 times less likely to receive intraoperative narcotics (odds ratio = 0.38; p = 0.031). However, there were no significant associations between TIVA and MME, either intraoperatively or postoperatively, or postoperative narcotic use (Table 3).

**Table 3:** Multivariate regression. P-values <0.05 are in bold. Controlling for age, sleep apnea, diabetes, asthma, GERD, and hypertension. TIVA: total intravenous anesthesia; MME: morphine milligram equivalent; OR: operating room; POSS: Pasero opioid-induced sedation scale; GERD: gastroesophageal reflux disease

Intra/Postoperative outcome	TIVA β/odds ratio	95% confidence interval	P-value
First PACU POSS (β)	-0.22	-0.43 to -0.01	0.040
Intraoperative MME (β)	0.20	-0.69 to 1.10	0.658
Intraoperative narcotics (odds ratio)	0.38	0.15 to 0.89	0.031
Postoperative MME (β)	-1.63	-4.18 to 0.92	0.202
Postoperative narcotics (odds ratio)	0.58	0.30 to 1.08	0.093
Minutes in OR (β)	-14.85	-20.23 to -9.46	<0.001
Minutes in recovery (β)	-6.16	-12.58 to 0.26	0.337
Perioperative minutes (β)	-21.01	-29.63 to -12.39	<0.001

## Discussion

Overall there were few, but potentially important, differences between groups, with TIVA patients experiencing similar or improved intraoperative and recovery outcomes than those receiving IA. The TIVA group demonstrated significantly shorter OR time, overall perioperative time, lower intraoperative dexamethasone administration, and lower first PACU POSS scores. The decreased OR time, perioperative time, and first PACU POSS score also remained significant in multivariate analysis after controlling for confounding variables. The percentage of patients receiving intraoperative narcotics was not significantly different in univariate analysis, but was significant upon multivariate analysis, with patients receiving TIVA being less likely to receive intraoperative narcotics.

GA with IA is the gold standard anesthesia technique for a wide variety of surgical procedures and is typically safe and effective, although it is accepted that some percentage of patients will experience side effects such as nausea, vomiting, poorly controlled postoperative pain, and postoperative cognitive impairment [6,17]. Several studies from a variety of surgical specialties indicate that TIVA may improve the quality of recovery after surgery by mitigating some of the uncomfortable effects of IA [11,17-19]. In contrast to a study by Kim et al., there was no difference in postoperative nausea scores, pain scores, and opioids given in this study when comparing IA and TIVA [11]. The one postoperative clinical outcome where TIVA patients performed better in this study was the first postoperative POSS score, although the mean POSS score in both groups was within an acceptable range; POSS scores less than 3 do not require any additional monitoring or intervention [16]. The reasons for improved POSS scores are not clear, although TIVA using propofol has been shown to improve analgesia and anxiolysis [13,20,21].

Another notable finding of this study was the decreased operative time and overall perioperative time in the TIVA group. Increased operative time has been identified as an independent risk factor in postoperative complications following arthroscopic shoulder surgery, including RCR [22-25]. Agarwalla et al. found that a 15-minute increase in operative time in arthroscopic RCR increased the risk of several postoperative complications, including surgical site infection, pulmonary embolism, and extended hospital stay [26]. We found a decrease in operative time between groups, as patients receiving TIVA had a shorter OR time overall, although this did not result in a difference in postoperative complications in this study. Further, the decreased OR time led to a significantly shorter overall perioperative time in TIVA patients, even though the six-minute reduction in PACU time for TIVA patients did not reach statistical significance independently.

Intraoperative and postoperative analgesia are significant concerns in patients undergoing RCR, as this surgery is associated with significant postoperative pain that affects both short-term patient comfort and longer-term recovery [7]. Regional anesthesia, often in the form of an interscalene nerve block, combined with GA has proven very effective at managing postoperative pain in these patients [6-8,15]. It is not known whether the type of GA, when combined with regional anesthesia, influences postoperative pain and intra and postoperative opioid consumption. When comparing IA and TIVA in general surgery, patients receiving TIVA require fewer opioid medications in the perioperative period [27,28]. Similar results have been found in orthopedic spine surgery, with patients receiving TIVA requiring fewer opioids in the postoperative period [11,29]. In contrast, patients in this study received equivalent total MMEs intraoperatively and in the PACU, although patients receiving TIVA were less likely to get any opioid medication intraoperatively. We hypothesize that our results deviated from those of the general and spine surgery literature given the consistent use of regional anesthesia in this population, which resulted in relatively low use of opioid analgesia, as both groups received under 20 MMEs both intra and postoperatively, on average.

Collectively, the benefits of TIVA observed in this study, namely, decreased intraoperative narcotic utilization rates, postoperative sedation, OR time, and perioperative time, suggest this anesthetic approach may hold value in facilitating the rapid throughput of RCR patients. As arthroscopic procedures, including RCR, continue to shift to the ambulatory surgery center setting, an increased focus on factors influencing perioperative throughput has occurred [30-34]. While many factors that have been shown to influence throughput time, such as technical aspects of the repair and concomitant procedures performed, sex, and comorbidities, are not modifiable, anesthetic selection is within the control of the care team [32,35,36]. The overall perioperative time savings of approximately 21 minutes per patient observed in this study is not insignificant and could allow for the performance of additional cases during the operative day. Given this benefit and the similar complication rates observed in comparison to IA, TIVA appears to be an attractive anesthetic option, particularly in the ambulatory care setting.

Despite the potential benefits of TIVA, not all patients are appropriate candidates. Anesthetic underdosing resulting in awareness during anesthesia is more likely to occur using TIVA techniques, and patients with a history of anesthetic resistance are at increased risk for awareness [37-39]. Further, caution should be taken when utilizing TIVA in morbidly obese patients. Although commonly practiced, appropriately dosing TIVA agents to avoid intraoperative awareness is more challenging in this population, as excess fat may result in increased diffusion of highly lipid-soluble propofol from the plasma [40]. While the results of the current study highlight the potential benefits of TIVA, the ultimate decision for anesthetic technique must continue to be made on a patient-specific basis, based on the clinical judgment of the anesthesiologist.

This study does have several limitations. First, the study was performed at a single institution and therefore the findings may not be generalizable to the wider patient population. It is also a retrospective study and subject to an inherent selection bias, although we were able to utilize multivariate analysis to control for potentially confounding factors. However, it is possible that unmeasured confounders influenced our results, including surgeon and anesthesiologist variability. A strength of the study is the consistent use of interscalene peripheral nerve blocks, thus eliminating a potentially significant confounding factor. Finally, this study is limited in its evaluation of only intraoperative and immediate postoperative outcomes. Further study is required to evaluate whether TIVA administration is associated with differences in post-discharge pain and narcotic utilization and patient perception of the operative experience. Based on the positive results presented, we suggest a prospective randomized evaluation is warranted to generate high-quality evidence evaluating the effects of TIVA on RCR outcomes.

## Conclusions

In comparison to IA, TIVA appears to be a safe and effective anesthetic for patients undergoing arthroscopic RCR with regional nerve blocks. Given the observed benefits, including reduced intraoperative narcotic utilization, postoperative sedation, and throughput time, we suggest TIVA be considered as a potentially beneficial alternative to IA for this patient population. There was no significant difference between anesthesia types in postoperative nausea, postoperative pain scores, or complications which suggests at minimum TIVA is equivalent to IA.

## References

[1] Zhao J, Luo M, Pan J (2021). Risk factors affecting rotator cuff retear after arthroscopic repair: a meta-analysis and systematic review. J Shoulder Elbow Surg.

[2] Gagnier J, Bedi A, Carpenter J, Robbins C, Miller B (2021). A 5-year follow-up of patients treated for full-thickness rotator cuff tears: a prospective cohort study. Orthop J Sports Med.

[3] Gomoll AH, Katz JN, Warner JJ, Millett PJ (2004). Rotator cuff disorders: recognition and management among patients with shoulder pain. Arthritis Rheum.

[4] Mather RC 3rd, Koenig L, Acevedo D (2013). The societal and economic value of rotator cuff repair. J Bone Joint Surg Am.

[5] Kalthoff A, Sanda M, Tate P (2022). Peripheral nerve blocks outperform general anesthesia for pain control in arthroscopic rotator cuff repair: a systematic review and meta-analysis. Arthroscopy.

[6] Maurya I, Garg R, Jain VK, Iyengar KP, Vaishya R (2021). Perioperative anaesthetic considerations for rotator cuff repair surgeries: a current concept review. J Clin Orthop Trauma.

[7] Kwater AP, Hernandez N, Artime C, de Haan JB (2021). Interscalene block for analgesia in orthopedic treatment of shoulder trauma: single-dose liposomal bupivacaine versus perineural catheter. Local Reg Anesth.

[8] Koga R, Funakoshi T, Yamamoto Y, Kusano H (2020). Suprascapular nerve block versus interscalene block for analgesia after arthroscopic rotator cuff repair. J Orthop.

[9] Smith G, D'Cruz JR, Rondeau B, Goldman J (2023). General Anesthesia for Surgeons. https://www.ncbi.nlm.nih.gov/pubmed/29630251.

[10] Miller AL, Theodore D, Widrich J (2023). Inhalational Anesthetic. https://www.ncbi.nlm.nih.gov/pubmed/32119427.

[11] Kim SH, Ju HM, Choi CH, Park HR, Shin S (2021). Inhalational versus intravenous maintenance of anesthesia for quality of recovery in patients undergoing corrective lower limb osteotomy: a randomized controlled trial. PLoS One.

[12] Yoshimura M, Shiramoto H, Morimoto Y, Koga M (2022). Comparison of total intravenous with inhalational anesthesia in terms of postoperative delirium and complications in older patients: a nationwide population-based study. J Anesth.

[13] Folino TB, Muco E, Safadi AO, Parks LJ (2023). Propofol. https://www.ncbi.nlm.nih.gov/pubmed/28613634.

[14] Coetzee JF (2009). Total intravenous anaesthesia to obese patients: largely guesswork?. Eur J Anaesthesiol.

[15] Mandava NK, Sethi PM, Routman HD, Liddy N, Haidamous G, Denard PJ (2021). Opioid requirement after rotator cuff repair is low with a multimodal approach to pain. J Shoulder Elbow Surg.

[16] Davis C, Geik C, Arthur K (2017). A multisite retrospective study evaluating the implementation of the Pasero opioid-induced sedation scale (POSS) and its effect on patient safety outcomes. Pain Manag Nurs.

[17] Meng W, Yang C, Wei X, Wang S, Kang F, Huang X, Li J (2021). Type of anesthesia and quality of recovery in male patients undergoing lumbar surgery: a randomized trial comparing propofol-remifentanil total i.v. anesthesia with sevoflurane anesthesia. BMC Anesthesiol.

[18] Kim DH, Min KT, Kim EH, Choi YS, Choi SH (2022). Comparison of the effects of inhalational and total intravenous anesthesia on quality of recovery in patients undergoing endoscopic transsphenoidal pituitary surgery: a randomized controlled trial. Int J Med Sci.

[19] Liu TC, Lai HC, Lu CH, Huang YS, Hung NK, Cherng CH, Wu ZF (2018). Analysis of anesthesia-controlled operating room time after propofol-based total intravenous anesthesia compared with desflurane anesthesia in functional endoscopic sinus surgery. Medicine (Baltimore).

[20] Sahinovic MM, Struys MM, Absalom AR (2018). Clinical pharmacokinetics and pharmacodynamics of propofol. Clin Pharmacokinet.

[21] Schraag S, Pradelli L, Alsaleh AJ (2018). Propofol vs. inhalational agents to maintain general anaesthesia in ambulatory and in-patient surgery: a systematic review and meta-analysis. BMC Anesthesiol.

[22] Shields E, Thirukumaran C, Thorsness R, Noyes K, Voloshin I (2015). An analysis of adult patient risk factors and complications within 30 days after arthroscopic shoulder surgery. Arthroscopy.

[23] Martin CT, Gao Y, Pugely AJ, Wolf BR (2013). 30-day morbidity and mortality after elective shoulder arthroscopy: a review of 9410 cases. J Shoulder Elbow Surg.

[24] Hill JR, McKnight B, Pannell WC (2017). Risk factors for 30-day readmission following shoulder arthroscopy. Arthroscopy.

[25] Heyer JH, Kuang X, Amdur RL, Pandarinath R (2018). Identifiable risk factors for thirty-day complications following arthroscopic rotator cuff repair. Phys Sportsmed.

[26] Agarwalla A, Gowd AK, Yao K (2019). A 15-minute incremental increase in operative duration is associated with an additional risk of complications within 30 days after arthroscopic rotator cuff repair. Orthop J Sports Med.

[27] Elbakry AE, Sultan WE, Ibrahim E (2018). A comparison between inhalational (desflurane) and total intravenous anaesthesia (Propofol and dexmedetomidine) in improving postoperative recovery for morbidly obese patients undergoing laparoscopic sleeve gastrectomy: a double-blinded randomised controlled trial. J Clin Anesth.

[28] Kim DH, Yun HJ, Park S, Leem JG, Karm MH, Choi SS (2020). Comparison between total intravenous anesthesia and balanced anesthesia on postoperative opioid consumption in patients who underwent laparoscopic-assisted distal gastrectomy. Medicine (Baltimore).

[29] Lin WL, Lee MS, Wong CS, Chan SM, Lai HC, Wu ZF, Lu CH (2019). Effects of intraoperative propofol-based total intravenous anesthesia on postoperative pain in spine surgery: comparison with desflurane anesthesia - a randomised trial. Medicine (Baltimore).

[30] Berlet GC, Weil LS Sr, Fooman A, Miller JM (2013). Contemporary trends in operating room efficiency. Foot Ankle Spec.

[31] Colvin AC, Egorova N, Harrison AK, Moskowitz A, Flatow EL (2012). National trends in rotator cuff repair. J Bone Joint Surg Am.

[32] Curry EJ, Logan C, Suslavich K, Whitlock K, Berkson E, Matzkin E (2018). Factors impacting arthroscopic rotator cuff repair operational throughput time at an ambulatory care center. Orthop Rev (Pavia).

[33] McLaren JM, Reynolds JA, Cox MM, Lyall JS, McCarthy M, McNoble EM, Petersen VR (2015). Decreasing the length of stay in phase I postanesthesia care unit: an evidence-based approach. J Perianesth Nurs.

[34] Small TJ, Gad BV, Klika AK, Mounir-Soliman LS, Gerritsen RL, Barsoum WK (2013). Dedicated orthopedic operating room unit improves operating room efficiency. J Arthroplasty.

[35] Boyd JA, Karas SG, Urchek RJ, Farley KX, Anastasio AT, Gottschalk MB (2020). Factors influencing operative time in arthroscopic rotator cuff repair: a comparison of knotless single-row vs. transosseous equivalent dual-row techniques. J Shoulder Elbow Surg.

[36] Stitz DJ, Guo AA, Lam PH, Murrell GA (2023). Determinants of operative time in arthroscopic rotator cuff repair. J Clin Med.

[37] Zhang C, Xu L, Ma YQ (2011). Bispectral index monitoring prevent awareness during total intravenous anesthesia: a prospective, randomized, double-blinded, multi-center controlled trial. Chin Med J (Engl).

[38] Morimoto Y, Nogami Y, Harada K, Tsubokawa T, Masui K (2011). Awareness during anesthesia: the results of a questionnaire survey in Japan. J Anesth.

[39] Pandit JJ, Andrade J, Bogod DG (2014). 5th National Audit Project (NAP5) on accidental awareness during general anaesthesia: summary of main findings and risk factors. Br J Anaesth.

[40] Al-Rifai Z, Mulvey D (2016). Principles of total intravenous anaesthesia: practical aspects of using total intravenous anaesthesia. BJA Educ.

